# Webster’s Dictionary

**Published:** 1849-01

**Authors:** 


### WEBSTER'S DICTIONARY. (Unabridged,)

Why notice Webster's Dictionary in a *medical* journal, some reader may be tempted to inquire? Our answer is, that a dictionary is an indispensable part of every physician's library, and the question is often asked us, whose shall I buy? Unhesitatingly we say, *Websters*—the unabridged edition, revised and enlarged by Chauncey A. Goodrich, and published at Springfield, Mass., for which H. W. Derby & Co., of Cincinnati, are the Western agents. We have a copy of the magnificent work on our table from the bookstore of John V. Cowling, in this city. Take it for all in all, our opinion is that no dictionary extant can compare with it.

